# Determining the influence of different linking patterns on the stability of students’ score adjustments produced using Video-based Examiner Score Comparison and Adjustment (VESCA)

**DOI:** 10.1186/s12909-022-03115-1

**Published:** 2022-01-17

**Authors:** Peter Yeates, Gareth McCray, Alice Moult, Natalie Cope, Richard Fuller, Robert McKinley

**Affiliations:** 1 School of Medicine, David Weatherall Building, Keele University, Keele, Staffordshire, ST5 5BG UK; 2 Fairfield General Hospital, Northern Care Alliance NHS Foundation Trust, Rochdale Old Road, Bury, BL9 7TD Lancashire UK; 3 Christie Education, Christie Hospitals NHS Foundation Trust, Wilmslow Rd, Manchester, M20 4BX UK

**Keywords:** Assessment, Objective Structured Clinical Exams, Assessor variability, Distributed assessments, Test Equating, Many Facet Rasch Modelling, Psychometrics

## Abstract

**Background:**

Ensuring equivalence of examiners’ judgements across different groups of examiners is a priority for large scale performance assessments in clinical education, both to enhance fairness and reassure the public. This study extends insight into an innovation called Video-based Examiner Score Comparison and Adjustment (VESCA) which uses video scoring to link otherwise unlinked groups of examiners. This linkage enables comparison of the influence of different examiner-groups within a common frame of reference and provision of adjusted “fair” scores to students. Whilst this innovation promises substantial benefit to quality assurance of distributed Objective Structured Clinical Exams (OSCEs), questions remain about how the resulting score adjustments might be influenced by the specific parameters used to operationalise VESCA. Research questions, How similar are estimates of students’ score adjustments when the model is run with either: fewer comparison videos per participating examiner?; reduced numbers of participating examiners?

**Methods:**

Using secondary analysis of recent research which used VESCA to compare scoring tendencies of different examiner groups, we made numerous copies of the original data then selectively deleted video scores to reduce the number of 1/ linking videos per examiner (4 versus several permutations of 3,2,or 1 videos) or 2/examiner participation rates (all participating examiners (76%) versus several permutations of 70%, 60% or 50% participation). After analysing all resulting datasets with Many Facet Rasch Modelling (MFRM) we calculated students’ score adjustments for each dataset and compared these with score adjustments in the original data using Spearman’s correlations.

**Results:**

Students’ score adjustments derived form 3 videos per examiner correlated highly with score adjustments derived from 4 linking videos (median Rho = 0.93,IQR0.90–0.95,*p* < 0.001), with 2 (median Rho 0.85,IQR0.81–0.87,*p* < 0.001) and 1 linking videos (median Rho = 0.52(IQR0.46–0.64,*p* < 0.001) producing progressively smaller correlations. Score adjustments were similar for 76% participating examiners and 70% (median Rho = 0.97,IQR0.95–0.98,*p* < 0.001), and 60% (median Rho = 0.95,IQR0.94–0.98,*p* < 0.001) participation, but were lower and more variable for 50% examiner participation (median Rho = 0.78,IQR0.65–0.83, some ns).

**Conclusions:**

Whilst VESCA showed some sensitivity to the examined parameters, modest reductions in examiner participation rates or video numbers produced highly similar results. Employing VESCA in distributed or national exams could enhance quality assurance or exam fairness.

## Background

Assessment of practical skills in health professionals’ education relies on observation by examiners of trainees performing clinical tasks [1]. Ensuring that different examiners judge performances to consistent standards is critical to the chain of validity evidence [2, 3], particularly when assessments are conducted for high stakes assessments such as graduation, licencing or accreditation to a speciality [4]. Decades of research have demonstrated the perennial observation of significant inter-examiner variability[5–7] in both informal [8, 9] and standardized assessments [10], which has persisted despite targeted interventions based on examiner training [11, 12]

These issues may be compounded by the scale of modern high-stakes standardised examination systems which typically involve several parallel, non-overlapping circuits of practical exams in which unique groups of students meet comparatively or totally unique groups of examiners. These are sometimes distributed in space and time within the same institution and sometimes distributed across large geographical areas. These “fully-nested” designs (i.e. no cross-over between the students seen by different groups of examiners) pose difficulties for measurement as data from different groups are unlinked. The purpose of this paper is to provide additional scrutiny to a recent innovation which aims to target this situation.

High-stakes performance assessments in the healthcare professions are typically conducted via Objective Structured Clinical Exams (OSCEs) [13], in which students rotate around several timed, standardised simulated clinical tasks, known as “stations”. The simulated clinical tasks often involve interacting with an actor (or “simulated patient”) who portrays a scripted illness. Students may be expected to perform a consultation, a simulated procedure or physical examination. Students’ performances are judged by either a trained examiner who observes the performance or by the simulated patient who scores the performance in addition to portraying the patient role. Examiners differ between stations, so students encounter several examiners during the exam. High stakes decisions are made by aggregating scores across all stations which reduces the influence of individual examiner variability. Despite this, a number of prior studies suggest that important differences may occur between examiners in different parallel circuits of the exam[14] or between sites [15], accounting for as much as 15.7% [16] and 17.1% [17] of score variance.

Despite the potential implications of this variability between groups of examiners (referred to as “examiner-cohorts”) [14], these effects are rarely examined as fully nested assessment designs mean they cannot be readily investigated with conventional psychometric analyses. To address this issue, we have previously developed a procedure called Video-based Examiner Score Comparison and Adjustment (VESCA) [18]. This procedure has 3 steps: 1/ a small subset (4–6 out of > 100) of students are filmed on all stations as they complete their live OSCE performances; 2/ examiners from all separate groups of examiners (i.e. different examiner-cohorts) voluntarily score between 2 & 4 station-specific (i.e. showing the same task they have examined) video performances in addition to completing their live examining duties. Examiners from all examiner cohorts collectively score the same pool of video performances, so the resulting scores for these videos link the examiners and students in otherwise unlinked data; 3/ the newly linked data is analysed using Many Facet Rasch Modelling [19] to determine the influence of examiner-cohorts on scores and to provide adjusted “fair scores” for each student.

Across 2 studies which both used VESCA, our findings have suggested that examiner-cohort effects may be important, with the difference between the highest and lowest scoring examiner-cohorts equivalent to greater than one standard deviation of students’ ability (Cohen’s d of 1.06(18) and 1.30 [20] respectively). Adjusting students’ scores accordingly altered 40% of students’ scores by at least *d* = 0.5, whilst adjustments might have altered pass fail decisions for up 16% of students. These findings suggest that if VESCA were employed in high stakes assessments it could potentially have significant implications for a subset of students. Both papers presented a range of data to support the appropriateness of the data for Many Facet Rasch analysis: data dimensionality, scale characteristics, fit of the data to the model and equivalence of scores between live and video performances. All of these were supportive.

Despite these reassurances, another consideration is important. VESCA relies on linking examiner-cohorts through a comparatively small sample of video performances. In Yeates et al.’s 2019 study, [18] examiners were asked to score just 2 comparison videos. By contrast, examiners were asked to score 4 comparison videos in Yeates et al.’s 2021 study [20]. Additionally, examiner participation was voluntary in both studies and resulted in 71% and 76% of examiners participating, meaning that scores for video performances comprised 8% and 20% of the overall datasets respectively. As both these factors (number of linking videos and examiner participation rates) have the potential to influence the model and therefore students’ adjusted scores, understanding their impact is important. Moreover, as increasing the number of linking videos produces an important additional burden in terms of time and organisation impact, which may further serve to reduce examiner participation, understanding the extent to which they both influence students’ score adjustments is important.

Guidance suggests that adequate linkage can be achieved using a Many Facet Rasch Model with as little as 7% linking data [21]. Empirical literature in support of this claim is, however, very sparse. Myford and Wolfe [22] embedded common linking performances within assessments of English language skills and found that whilst including linking performances had a substantial influence on estimates of examiner stringency, there was no consistent relationship between the number of linking performances and the quality of linkage achieved. Consistently Wind and Jones [23], using simulated data, showed that estimates made using either 3, 6 or 8 linking performances per examiner were extremely highly correlated (0.99–1.0). In contrast, Wind et al. [24] showed that the consistency of performance estimates deteriorated progressively as the number of linking performances was reduced. All of these studies address linking of individual examiners rather than examiner-cohorts and none determined the influence of reduced examiner participation in the linking exercise. Consequently, the impact of these factors on the adjusted scores produced by the VESCA intervention remains difficult to predict.

As it is critical to the defensible use of VESCA within OSCEs to understand how much the intervention conditions might have influenced scores adjustments, in this present study we aimed to examine the sensitivity of score adjustments to the number of linking videos and to examiner participation rates. On that basis, using the dataset from Yeates et al. [20], we asked the following research questions.

How similar are estimates of students’ score adjustments when the model is run with either 4, 3, 2 or 1 comparison videos per participating examiner?

How similar are estimates of students’ score adjustments when the model is run with reduced numbers of participating examiners?

## Methods

We addressed these questions by means of secondary data analysis, using the data from Yeates et al. 2021 [20] All analyses were within the purview of the ethical application for Yeates et al. 2021 [20] , so no additional ethical approval was sought.

### Dataset

The dataset comprised scores from 113 Year 3 medical students sitting an end of year OSCE exam which contributed to their progression into the next academic year. The OSCE comprised twelve, 10-min stations, with each student completing all 12 stations. Owing to student numbers, students were distributed across 1 of 4 parallel (ostensibly identical) circuits. Procedures were repeated for both the morning and afternoon, but whilst the stations remained the same all day, most examiners only examined for a morning or afternoon. Consequently, there were 8 separate examiner-cohorts (am & pm cohorts, in each of the 4 parallel circuits). In addition to scoring the performances of 12–15 “live” students, examiners were additionally invited to score 4 video performances of students. Each participating examiner viewed videos of students performing the same station tasks as the station which they had examined, although (for most examiners) the videos showed students they had not previously examined. For labelling purposes these videos were denoted videos A-D. All videos were filmed during the first part of the morning session. Participating afternoon examiners scored videos via tablet computers in gaps between live students (termed “embedded”), whilst participating morning examiners scored videos via the internet over the 3 weeks after the exam (termed “internet”). Examiners scored student performances in several domains, using Likert scales, which summed to give a total score with possible range 6–27 for each station. As reported by Yeates et al. 2021 [20], there was no systematic difference between live and video performances for a subset of examiners who scored the same students in both formats; and no difference between scores for embedded and internet scoring modalities. Seventy-six percent of examiners participated (*n* = 73), and scores given to comparison videos comprised 17.7% of the total data. All data were labelled with the anonymous identifiers denoting the student, examiner, examiner cohort, and labels for the station and the sequence of each performance within the performances seen by each examiner cohort.

Data in Yeates et al. [20] were analysed using a Many Facet Rasch Model [19], using the software package FACETS [25]. Whilst only a small number of volunteer students participated in videoing, the linkage achieved by their scores meant that FACETS [25] was able to produce adjusted “fair” scores (i.e., scores which adjust for differences between examiner cohorts via the linkage) for all students in the OSCE. Both the original unadjusted raw scores and the adjusted “Fair-scores” were analysed within the current study.

### Number of Comparison videos per participating examiner

To understand how the number of comparison videos influenced adjustments to students’ scores, we firstly calculated the “score adjustment” for each student by subtracting each student’s observed (raw) score from their adjusted “fair-score”. When this value was > 0 it indicated that this student’s score had been increased by the adjustment made by the MFRM, whereas when this value was < 0 it indicated that the student’s score had been decreased by the adjustment.

**Fig. 1:**
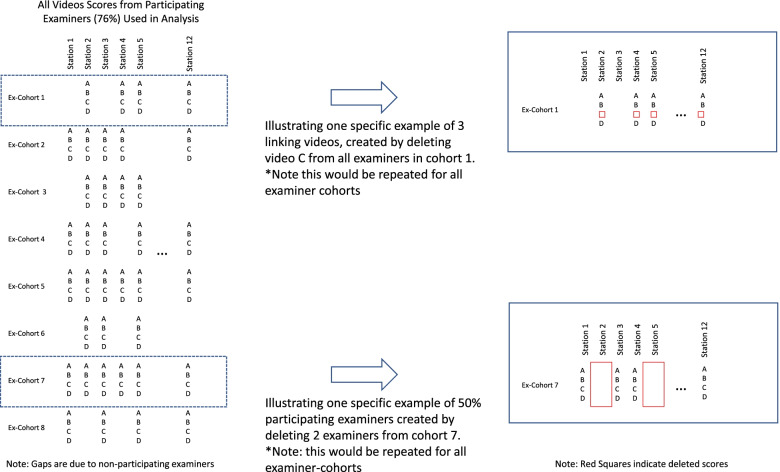
Illustration of manipulation of linking process. This figure Illustrates the procedures used to create fewer linking videos per participating examiner (top) and fewer participating examiners (bottom) by deleting videos from the original dataset

Next, we created numerous copies of the original data, but in each copy, we selectively removed video-score data so that (rather than there being 4 video scores for each participating examiner) there were either 3, 2 or 1 video scores per participating examiner. Data were removed from all participating examiners equally, and in each case the videos denoted with the same letter (i.e. A,B,C,D) were removed. Various permutations were available in terms of how this could be performed. For example, 3 videos can be achieved through 4 different permutations: ABC, ABD, ACD, BCD. Six permutations were available for 2 linking videos per station and 4 permutations were available for 1 linking video per station. Data were prepared for all of these permutations of 3, 2 and 1 linking video per station (total of 14 datasets). The process of video deletion to produce fewer linking videos per examiner is illustrated in Fig. 1.

The resulting datasets were then individually analysed using FACETS [25] to produce adjusted scores for each student from all 14 of the data sets (i.e. each permutation of 3,2 and 1 linking videos). For each of these datasets we then recalculated the score adjustment (adjusted score – raw score) for each individual student. Following this, we performed a Spearman’s correlation between the 1/ score adjustment for each student calculated when the analysis used all 4 linking videos and 2/ the score adjustment for each student when the analysis used a specific permutation of fewer videos. This process was repeated for all of the tested permutations of 3, 2 and 1 videos.

Next, we aimed to understand whether score adjustments are influenced by the choice of videos used in linking (i.e. whether linking via videos A&B would give different score adjustments than linking via videos C&D). To do this we compared the relationship between 1/ students’ score adjustment derived from non-overlapping combinations of videos (i.e. score adjustments derived using videos A&B vs score adjustments derived using videos C&D) with students’ score adjustments derived from partially-overlapping combinations of videos (i.e. score adjustments derived from using video A&B vs videos A&C). This was only possible for score adjustments derived using two linking videos as no other combinations of videos had both non-overlapping and partially overlapping permutations. We performed Spearman’s correlations between students’ score adjustments derived from all non-overlapping combinations of 2 videos (i.e. A&B vs C&D; A&C, vs B&D; A&D vs B&C) and all partially overlapping combinations of 2 videos (i.e. A&C vs A&B; B&C vs C&D etc.) and then compared the resulting correlation coefficients between conditions. We adjusted significance levels for all correlations using the Bonferroni correction [26] to avoid type 1 error. Analyses were conducted in SPSS v26 [27].

### Number of participating examiners

We examined the influence of reduced examiner participation on score adjustments using similar methods to those used for calculating score adjustments for fewer linking videos. The complete dataset was again copied multiple times, but instead of removing a specific number of videos from each participating examiner, we removed all video score data for a selected number of participating examiners, in order to model examiner participation rates of 70%, 60% and 50%. For each level of examiner participation, we repeated the analysis 5 times, removing different examiners each time. Examiner removal was performed by randomly ordering all examiners and then removing a specified number in order to achieve the required percentage participation required. These procedures gave 15 further datasets. The process of video deletion to reduce examiner participation is illustrated in Fig. 1.

The resulting datasets were again individually analysed using FACETs to produce fair scores for each student. Score adjustments were calculated for each student for each permutation. Spearman’s correlations were performed between 1/ the score adjustment for each student based on analysis using all participating examiners (76%) and 2/ the score adjustment for each student based on analysis using a specific permutation of fewer participating examiners. This was repeated for all 20 permutations of reduced participating examiners.

## Results

### Number of comparison videos per participating examiner

Fourteen distributions of students score adjustments were produced by the described analyses, with 113 cases in each. The range of students’ score adjustments varied across different distributions, but typically ranged from around -1.3 to 1.3 with means and medians close to 0. The range of students’ score adjustments tended to be higher for analyses based on fewer linking videos.

Spearman’s correlation coefficients between students score adjustments derived using all videos and different permutations of three videos ranged from rho = 0.87–0.97 with a median of 0.93(Inter Quartile Range 0.90–0.95). All were significant at the Bonferroni-corrected *p* = 0.001. Spearman’s correlation coefficients between students score adjustments derived using all videos and different permutations of two videos ranged from rho = 0.65–0.94, median = 0.85 (IQR0.81–0.87), all *p* < 0.001. Spearman’s correlation coefficients between students’ score adjustments derived using all videos and different permutations of one video ranged from rho = 0.36–0.91, median = 0.52 (IQR 0.46–0.64), all *p* < 0.001. See Table 1.

**Table 1:** Correlations between score adjustments derived from all linking videos (ABCD) and score adjustments derived from different permutations of fewer linking videos

	Correlation Coefficient (rho)	*p*
ABCD	1	-
3 linking videos per participating examiner		
ABC	0.91	0.000
ABD	0.97	0.000
ACD	0.87	0.000
BCD	0.95	0.000
Median	0.93	IQRs (0.90–0.95)
2 linking videos per participating examiner		
AB	0.94	0.000
AC	0.80	0.000
AD	0.88	0.000
BC	0.86	0.000
BD	0.84	0.000
CD	0.65	0.000
Median	0.85	IQRs (0.81–0.87)
1 linking video per participating examiner		
A	0.91	0.000
B	0.49	0.000
C	0.36	0.000
D	0.55	0.000
Median	0.52	IQRs (0.46–64)

Spearman’s correlation coefficients between students’ score adjustments derived from the three available non-overlapping combinations of two videos were: A&C vs B&D rho = 0.41, A&B vs C&D rho = 0.58, A&D vs B&C rho = 0.62, median rho = 0.58 (IQR 0.50–0.60), all *p* < 0.001. Conversely Spearman’s correlation coefficients between students’ score adjustments derived from the twelve partially overlapping combinations of two videos ranged from rho = 0.42 – 0.92, median 0.75 (IQR 0.63–0.78), all *p* < 0.001. See Table 2.

**Table 2:** Spearman’s correlations (with associated p values) between score adjustments derived from different pairs of overlapping (white background) or non-overlapping (grey background) linking videos

	AB	AC	AD	BC	BD	CD
AB	1	0.846	0.922	0.789	0.774	0.579
		< 0.001	< 0.001	< 0.001	< 0.001	< 0.001
AC		1	0.778	0.73	0.412	0.649
			< 0.001	< 0.001	< 0.001	< 0.001
AD			1	0.621	0.762	0.625
				< 0.001	< 0.001	< 0.001
BC				1	0.627	0.425
					< 0.001	< 0.001
BD					1	0.535
						< 0.001
CD						1

### Proportion of participating examiners

Twenty distributions of students’ score adjustments were produced by the described analyses, each with 113 cases. Ranges of score adjustment and distributions were similar to those for fewer combinations of videos. Spearman’s correlation coefficients between students’ score adjustments derived from all (76%) participating examiners and 70% participating examines ranged from rho = 0.86–0.99, median = 0.97 (IQR 0.95–0.98), all *p* < 0.001. Spearman’s correlation coefficients between students’ score adjustments derived from all participating examiners and 60% examiner participation ranged from rho = 0.92–0.98, median = 0.95 (IQR 0.94–0.98), all *p* < 0.001. Spearman’s correlation coefficients between students’ score adjustments derived from all participating examiners and 50% examiner participation ranged from rho = 0.29 (non-significant) to 0.93 (*p* < 0.001), median = 0.78 (IQR 0.65–0.83), all *p* < 0.001 except the lowest value. See Table 3.

**Table 3:** Correlations between score adjustments derived from using linking scores provided by all participating (76%) of examiners and score adjustments derived from linking scores provided by different permutations of fewer participating examiners

	Correlation Coefficient rho	*p*
All participating examiners (76%)	1.000	-
Score adjustments derived from 70% examiner participation		
Combination A	0.99	0.000
Combination B	0.95	0.000
Combination C	0.86	0.000
Combination D	0.98	0.000
Combination E	0.97	0.000
Median	0.97	IQR (0.91–0.99)
Score adjustments derived from 60% examiner participation		
Combination A	0.94	0.000
Combination B	0.98	0.000
Combination C	0.92	0.000
Combination D	0.95	0.000
Combination E	0.98	0.000
Median	0.95	IQR (0.93–0.98)
Score adjustments derived from 50% examiner participation		
Combination A	0.65	0.000
Combination B	0.78	0.000
Combination C	0.83	0.000
Combination D	0.29	0.002
Combination E	0.93	0.000
Median	0.78	IQR (0.47–0.88)

## Discussion

### Summary of findings with theoretical interpretation

As might be expected, given the degree of overlap in linking material, score adjustments based on 3 linking videos correlated very highly with score adjustments based on all videos, whereas adjusted scores derived from 2 and 1 linking videos were both progressively weaker and showed wider variations in correlation coefficients. Consequently we may assert that had the data used in this study been derived using just two linking videos (as was the case in Yeates et al., 2019 [18] rather than four linking videos, students’ score adjustments would (on average) have correlated at rho = 0.85. As a result (and as anticipated), whilst the VESCA procedure does produce broadly similar results with fewer linking videos, it appears to show some sensitivity to the degree of linkage achieved. Correlations between students’ score adjustments derived from two independent linking videos (ie. A&B vs C&D) were lower than the correlations between score adjustments derived from two partially overlapping linking videos (i.e. A&B vs A&D). This tends to suggest that (at least when there are two linking videos) scores adjustments show some sensitivity to the choice of videos used to perform linkage as well as their number.

Score adjustments derived from analyses using fewer examiners showed very high correlations between all examiners and both 60% and 70% examiner participation. Conversely for 50% of participating examiners the range of correlations was wide, from rho = 0.29 (non-significant) to 0.93 (highly significant). This suggests that whilst the 60% and 70% levels of examiner participation gave consistently highly similar score adjustments, whereas at 50% examiner participation the method had become somewhat less stable.

Collectively these findings suggest that using 3 linking videos per station, or 4 videos per station with only 60–70% examiner participation, would have produced very similar student score adjustments to those achieved with all participating examiners (76%) scoring 4 videos.

From a theoretical perspective, the purpose of the VESCA innovation is to link otherwise unlinked groups of examiners, in order to directly compare their stringency / leniency within a shared frame of reference. This in turn enables adjustment of students’ scores in a manner which is intended to negate the influence of the group of examiners a particular student encountered, thereby reducing construct-irrelevant variance[18]. Whilst Many Facet Rasch Modelling is capable of using limited linkage within a dataset to place all elements within each facet on a common scale, and thereby iteratively calculating these influences within a common frame of reference [19], the linkage pattern which is adopted should ideally not contribute further to error variance.

Linacre has suggested that productive linkage within a Many Facet Rasch Model can be achieved (at least theoretically) using as little as 7% linkage [21], by adopting an optimally efficient linkage pattern. Nonetheless, this claim is made on the basis that a link of at least 1 score exists between all raters, candidates and tasks within the dataset, and does not explore the stability of the resulting estimates. In our dataset, 50%, 60% and 70% examiner participation rates (all scoring 4 videos) represented somewhat greater linkage at 12.4%, 14.5 and 16.5% linkage respectively (i.e. the percentage of the total dataset comprised of examiners’ scores for videos). The observation of less than perfect (albeit high) correlations within our findings underscores Linacre’s assertion that the design of the linkage pattern is critical to its success.

Whilst, as noted in the background, prior literature on this topic is very sparse, our findings stand at odds with two of the three cited studies. Wind and Jones [23] showed near perfect correlations between adjusted scores for estimates linked by either 8, 6 or 3 performances. This tended (counter-intuitively) to suggest that the number of linking performances was irrelevant. Whilst this appears to contradict our findings, as we also found very high correlations between adjusted scores derived from 3 and 4 linking videos. This might lead to speculation about the potential for a threshold at around 3 to 4 linking performances above which additional performances add little more stability to estimates. This suggestion would require further empirical work to support. By contrast, Myford and Wolfe [22] found that poor linkage could occur with 1 performance or 6; other factors appeared to determine the strength of linkage which they were unable to completely explain. They noted that the standard of the performance influenced linkage, with strong performances tending to show greater strength of linkage than weak performances. Both Myford and Wolfe [22] and Wind and Jones [23] showed that “noisy” data (i.e. data with poor fit to the Rasch model) achieved stronger linkage than “clean” (i.e. well-fitting) data. The reasons for this counter-intuitive observation are unclear. Yeates et al. [20] reported very good fit of the examiner-cohort data to the Many Facet Rasch Model which may partly explain the difference in observations. Lastly, as mention in the background, prior work has linked examiners as individuals whereas Yeates et al. [18, 20] have linked groups of examiners (i.e. examiner cohorts). This choice was made because the Many Facet Rasch Model was unable to provide estimates of individual examiner’s ability in either dataset. This appears to have been because examiner participation was less than 100%. It may be that modelling examiner-cohorts rather than individual examiners confers greater sensitivity to the linkage pattern.

### Practical implications

These data suggest some sensitivity of VESCA to the linkage strategy which is adopted when the intervention is employed. As this influence is likely to vary across contexts, it may be desirable to determine the sensitivity of adjusted scores to different linkage patterns within a given context and interpret results in the light of this variability. Notably, this is less likely to influence estimates of examiner cohort effects, so if VESCA is used purely for quality assurance purposes, the resulting influence of the linkage pattern on estimates is likely to be less important. Further work is required to establish whether general principles regarding the number of videos or percentage examine participation rates can be established to guide choices in practice, and to determine the overall accuracy of adjusted scores under various conditions. Implementing VESCA produces an additional workload for institutions and further development to streamline the pragmatics of filming and video scoring procedures may help to ensure that VESCA is sufficiently feasibility for institutions to easily realise its benefits in practice. Whilst videoing in OSCEs has the potential to disrupt students’ performances, prior research has demonstrated that as long as described principles are thoughtfully applied, videoing has little or no impact on students’ OSCE performances [30]

### Limitations and future research

The main limitations of this study emanate from the secondary dataset on which the study was based. We have used the score adjustments produced within the original study as a reference point for comparison but cannot exclude the potential that better estimates of students’ performance might have been obtained had the score adjustments been obtained from analyses based on greater numbers of linking videos or a higher proportion of participating examiners.

Our method in this study relied on deletion of video score data, which mimicked a situation where examiners had scored fewer linking videos or fewer examiners had participated. This approach assumed that the judgements which produced the scores which remained were unaffected by the judgements which produced the scores we deleted. As prior work has shown that sequentially judged performances influence each other [28, 29], this assumption may not always hold true, which constitutes a limitation of the method.

Whilst it may ultimately only be possible to understand these research questions using data simulation, this study has the benefit of providing insight into these issues on empirical grounds without the need for assumptions about the underlying causation of variability. Whilst findings from this study do not implicitly generalise to other settings in which VESCA may be used, the design of the OSCE in this instance was very typical of undergraduate OSCE designs and some transferability of the findings is likely. Regardless, further empirical work is needed to determine whether general principles for VESCA’s use can be established across a broader context.

## Conclusions

Video-based Examiner Score Comparison and Adjustment (VESCA) is a novel method of enhancing equivalence in distributed Objective Structured Clinical Exams which could be used to quality assure the fairness of national or widely distributed exams. Within the data examined, whilst the VESCA method showed some sensitivity to both the number of linking videos and examiner participation rates, modest reductions in examiner participation rates or the number of linking videos appear to produce highly similar results.

## Data Availability

The datasets generated and/or analysed during the current study are not publicly available due to restrictions placed on the use of data by the host institution (Keele School of Medicine) but are available from the corresponding author on reasonable request.

## Abbreviations

OSCE: Objective Structured Clinical Exam
VESCA: Video-based Examiner Score Comparison and Adjustment
